# Body in mind

**DOI:** 10.3389/fpsyg.2015.00056

**Published:** 2015-01-30

**Authors:** Alexander Jones, Bettina Forster

**Affiliations:** ^1^Department of Psychology, Middlesex University LondonLondon, UK; ^2^Department of Psychology, City University LondonLondon, UK

**Keywords:** body in mind, tactile, touch, somatosensory, attention, Somatosensory Evoked Potentials (SEP)

The sense of touch is imperative in cognition, development, and for how we perceive the world and interact with others. The skin envelopes our body and is our largest organ providing us with information about our immediate environment. In addition to this “surface” information, our somatosensory system also processes information regarding body position and internal bodily states. The importance of processing information to our body is unlikely to be disputed, however, relatively little research has focused on the somatosensory system and the body sense as compared to vision and audition. The last two decades have however seen an increased interest in the body sense from a range of different perspectives, using different methodologies which this research topic aimed to reflect.

The broad range of unanswered research questions relating to the body is also mirrored by the contributions in this research topic. We are constantly bombarded with tactile information, such as from the clothes on our body, or the chair we sit upon, yet we are able to process only what is relevant to us. Mechanisms of selective attentions help to prioritize, predict and select information relevant to the situation and to guide our behavior. Selectively attending to a location on the body has repeatedly shown to enhance processing of stimuli at attended over unattended location (e.g., Jones and Forster, 2014). In this research topic, Tamè et al. (2014) showed that detection of a tactile stimulus is dependent upon concurrent presented stimuli which may act as a detection mask, even though the mask is presented at different fingers or hands. Somatosensory information is not typically presented in isolation but the sense of touch is also integrated with other modalities (for an overview see Calvert et al., 2004). Using event-related potentials (ERPs), Staines et al. (2014) showed how visual information relevant to movements modulates somatosensory processing. In a comprehensive review, Heed and Azañón, 2014) explored the findings of how we localize touch to our body. Touch can be localized both using somatotopic but also external coordinates. That is, where on the skin was the stimulus, or where based on external spatial co-ordinates did the tactile stimulus appear. Their overview focuses on how temporal order judgment (TOJ) tasks have been a fruitful paradigm to investigate tactile spatial processing. External and somatotopic maps were also shown, using ERPs, to be affected by where vision is directed (Gherri and Forster, 2014).

Observing others being touched as well as the social aspect of touch are important parts of everyday life and this has also recently attracted increased research interest. In particular, in this research topic Gillmeister (2014) introduced a new behavioral paradigm to measure how observing touch on another person's hand influences the perception of one's own touch. Moreover, Farmer et al. (2014) showed how induced changes in body ownership through observation of touch applied to another person's hand of a different skin color can modulate social attitudes. A similar emerging and interesting research area, briefly covered in this research topic, is how we use interoceptive cues and how the sense of self develops (Sel, 2014). Last but not least, a study investigating the touch and weight perception on aesthetic appreciation points to an important role of one's own body perception in aesthetic perception (Yamada et al., 2014). Together, these recent, seemingly divers studies could fall under the term embodied cognition which now encompasses seemingly diverse fields of psychology highlighting the influence of the body in mind on cognition in general.

This research topic brings together both research from well-established paradigms and research areas as well as highlighting how the body and the sense of touch is also being explored in new ways. The contributions here show there are a broad range of perspectives and questions to be addressed when processing stimuli to and within our body. The increased interest in the sense of touch over the last decades will hopefully continue as much still remains to be explored.

## Conflict of interest statement

The authors declare that the research was conducted in the absence of any commercial or financial relationships that could be construed as a potential conflict of interest.
